# *Ex vivo* assessment and simulation to guide cefepime-taniborbactam dosing recommendations for patients receiving continuous renal replacement therapy

**DOI:** 10.1128/aac.00061-25

**Published:** 2025-05-05

**Authors:** Aliaa Fouad, Cole McGrath, Ecem Buyukyanbolu, Joseph L. Kuti

**Affiliations:** 1 Center for Anti-Infective Research and Development, Hartford Hospital 23893, , Hartford, Connecticut, USA; Providence Portland Medical Center, Portland, Oregon, USA

**Keywords:** pharmacokinetics, pharmacodynamics, renal impairment, antibiotic dosing

## Abstract

Cefepime-taniborbactam dosing in patients undergoing continuous renal replacement therapy (CRRT) is unknown. We employed an *ex vivo* CRRT model to characterize transmembrane clearance (CL_TM_) and derive optimal dosing regimens for patients supported by CRRT. CL_TM_ was determined in CVVH and CVVHD modes using the Prismaflex ST150 and HF1400 hemofilter sets. Samples were collected over 60 minutes to determine cefepime and taniborbactam concentrations at increasing effluent flow rates (ER). Sieving (SC) and saturation (SA) coefficients were measured and used to calculate CL_TM_. Multiple linear regression determined cefepime and taniborbactam CL_TM_ as a function of ER, hemofilter, and mode. An established population pharmacokinetic model was integrated with the CL_TM_, and a 1,000 patient Monte Carlo Simulation was conducted to determine exposures of potential dosing regimens for pneumonia. The overall mean ± SD SC/SA across CRRT modes, hemofilters, and ERs were 1.13 ± 0.08 and 1.03 ± 0.07 for cefepime and taniborbactam, respectively. ER was the primary driver (*P* < 0.001) of CL_TM_ for both drugs. For ER <3.5 L/h, cefepime and taniborbactam 1 g–0.25g q8h and 2 g–0.5g q12h as 4 h infusions achieved high probability of pharmacodynamic target attainment while keeping area under the curve exposures consistent with the proposed dose in pneumonia in non-CRRT patients. For ER ≥3.5 L/h, the optimum regimen was cefepime and taniborbactam 2 g–0.5 g q8h as a 4 h infusion. When incorporated into a population pharmacokinetic model, these CL_TM_ data were used to propose dosing recommendations for cefepime and taniborbactam as a function of ER in patients undergoing CRRT.

## INTRODUCTION

The increasing prevalence of multidrug resistance among gram-negative bacteria is a significant global concern that necessitates the development of new antibiotics (1). β-Lactamases play a significant role in reducing the effectiveness of current β-lactam antibiotics to Gram-negative organisms (2). Taniborbactam, a bicyclic boronate β-lactamase inhibitor that demonstrates strong inhibitory activity against both serine- and metallo-β-lactamases is currently being developed in combination with cefepime to offer a treatment option for patients with complicated urinary tract infections as a 2 hour infusion and hospital-acquired bacterial pneumonia (HABP) and ventilator-associated bacterial pneumonia (VABP) as a 4 hour infusion caused by drug-resistant gram-negative bacteria, particularly carbapenem-resistant Enterobacterales (CRE) and carbapenem-resistant *Pseudomonas aeruginosa* (CRPA) (3–7).

Optimal dosing is paramount in achieving safe and successful exposures for any antibiotic. The proposed dosing regimen for cefepime-taniborbactam in patients with normal kidney function is 2 g of cefepime and 0.5 g of taniborbactam co-administered every 8 hours (q8h) with each dose infused over 4 hours for the treatment of HABP and VABP. However, dose adjustments for patients with reduced renal function are required since both cefepime and taniborbactam are primarily removed through glomerular filtration and are excreted unchanged in the urine (8). While the pharmacokinetics of cefepime and taniborbactam have been determined in patients receiving intermittent hemodialysis, pharmacokinetics and dosage recommendations in patients receiving continuous renal replacement therapy (CRRT) are still needed.

CRRT is the most common treatment prescribed to manage severe acute kidney injury (AKI) in the Intensive Care Unit (9). It provides more precise volume control, gradual correction of metabolic abnormalities, and enhanced hemodynamic stability compared with intermittent hemodialysis (10). CRRT is very efficient at removing unwanted solutes, volume, as well as certain drugs through its semi-permeable membrane, which must be accounted for to continue to provide optimal and safe antibiotic exposures (11–13).

In the absence of clinical data to support dosing recommendations, *ex vivo* CRRT models can provide valuable information to determine adsorption and transmembrane clearance (CL_TM_) across effluent flow rates and CRRT scenarios (14–16). In this study, we aimed to characterize the adsorption, protein binding, and clearance (CL_TM_) of cefepime and taniborbactam in an *ex vivo* CRRT model using bovine whole blood and integrated the derived CL_TM_ values with established population pharmacokinetic models to propose dosing regimens for patients undergoing CRRT.

## RESULTS

### Degradation and adsorption studies

**Fig 1:**
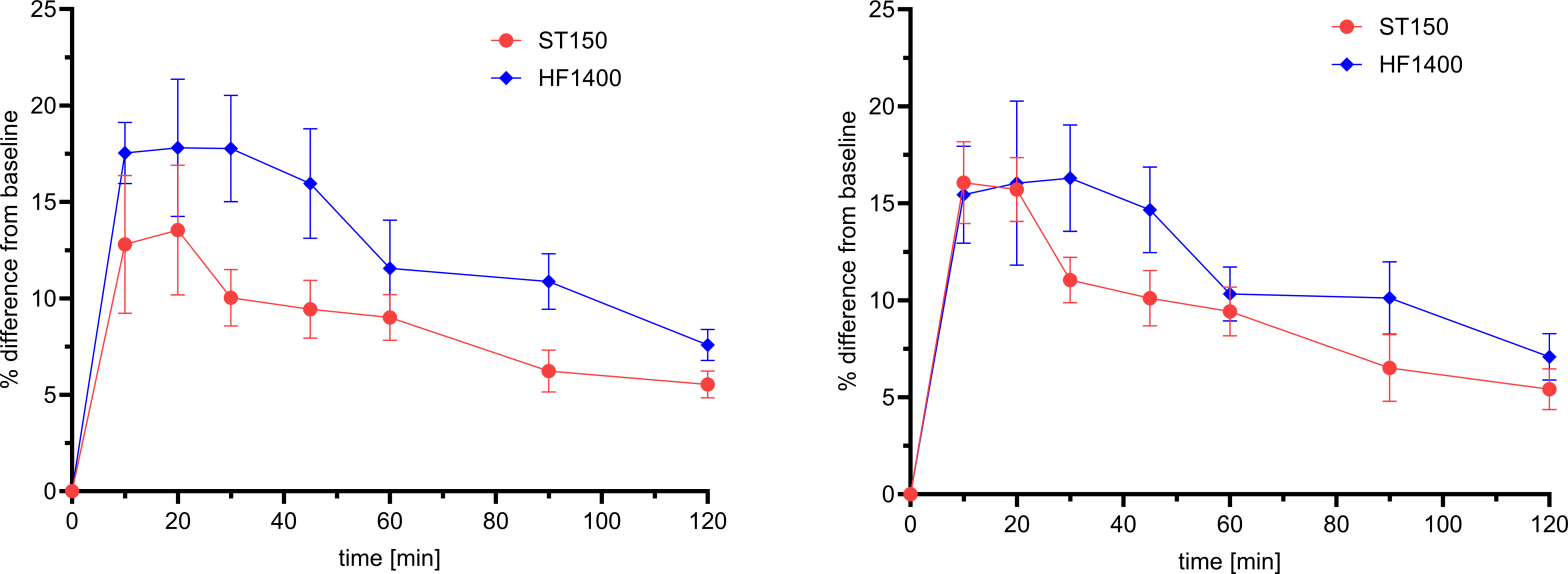
Summary results of cefepime (left), and taniborbactam (right) adsorption to ST150 and HF1400 hemofilters in whole bovine blood; data are mean and standard deviation of two duplicate runs for each filter and displayed as %adsorption from baseline concentrations.

Both cefepime and taniborbactam demonstrated mean degradation of <10% in bovine whole blood over 120 minutes, the maximum duration of all experiments (Table S1). Negligible adsorption (i.e., <20%) to the hemofilters was observed for cefepime and taniborbactam; however, adsorption was numerically higher to the polyarylethersulfone (PAES) HF1400 hemofilter compared with the acrylonitrile (AN69) ST150 hemofilter (Fig. 1; Table S2). Peak adsorption was observed at 20 minutes for both hemofilters. The observed degradation and adsorption are below that required to compensate for estimates CL_TM_ or to impact dosing regimens of cefepime-taniborbactam.

### Protein binding in bovine plasma

Cefepime protein binding in bovine whole blood was 0% across all experiments, while taniborbactam protein binding was 3.7 ± 3.8%. Protein binding was not concentration-dependent for either drug.

### Transmembrane clearance

The mean ± standard deviation (SD) starting concentration of cefepime and taniborbactam in the central reservoir of the *ex vivo* CRRT model was 66.8 ± 6.8 and 18.7 ± 1.8 µg/mL, respectively, which aligned with targeted peak concentrations in patients receiving 2 g–0.5 g q8h as a 4 hour infusion (70 and 20 µg/mL, respectively). Cefepime and taniborbactam were freely cleared during continuous veno-venous hemofiltration (CVVH) and continuous veno-venous hemodialysis (CVVHD) and using either hemofilter set with overall SC/SA of 1.13 ± 0.08 and 1.03 ± 0.07, respectively. Table 1 summarizes the SC and SA values for cefepime and taniborbactam by CRRT mode, hemofilter type, effluent flow rate, and point of replacement fluid dilution.

**TABLE 1:** Sieving (SC) and saturation coefficient (SA) of cefepime and taniborbactam during the *ex vivo* CRRT runs by mode, hemofilter, effluent rate, and point of dilution; values are mean ± SD of duplicate runs

CRRT mode	Cefepime		Taniborbactam	
	ST150	HF1400	ST150	HF1400
CVVH	SC	SC	SC	SC
2 L/h, 100% pre	1.08 ± 0.01	1.11 ± 0.00	0.99 ± 0.00	1.00 ± 0.01
2 L/h, 50/50%	1.08 ± 0.05	1.17 ± 0.05	1.00 ± 0.04	1.08 ± 0.05
3 L/h, 100% pre	1.15 ± 0.04	1.09 ± 0.00	1.08 ± 0.03	1.01 ± 0.03
3 L/h, 50/50%	1.07 ± 0.04	1.12 ± 0.07	0.97 ± 0.01	1.03 ± 0.05
4 L/h, 100% pre	1.11 ± 0.01	1.17 ± 0.02	1.00 ± 0.03	1.09 ± 0.05
4 L/h, 50/50%	1.06 ± 0.08	1.13 ± 0.02	0.97 ± 0.05	1.05 ± 0.01
CVVHD	SA	SA	SA	SA
2 L/h	1.17 ± 0.03	1.24 ± 0.16	1.08 ± 0.04	1.14 ± 0.19
3 L/h	1.10 ± 0.07	1.25 ± 0.08	1.03 ± 0.08	1.07 ± 0.16
4 L/h	0.97 ± 0.04	1.24 ± 0.02	0.92 ± 0.01	1.08 ± 0.08

### Impact of CRRT variables on CL_TM_

Multiple linear regression was performed to evaluate the influence of effluent flow rate, hemofilter type, and CRRT mode on cefepime and taniborbactam CL_TM_ (Table 2). While all variables were statistically significant for both drugs, the effluent flow rate resulted in the greatest slope coefficient. To simplify dosing regimens across various filter types and modes, additional multiple linear regression analyses were performed on effluent rate only. The final linear regressions were cefepime CL_TM_ (L/h) = 0.41 + (0.86 × effluent flow rate, L/h) (*P* < 0.0001); taniborbactam CL_TM_ (L/h) = 0.38 + (0.79 × effluent flow rate, L/h) (*P* < 0.0001). These simplified regressions were used to perform all Monte Carlo simulations for dosage selection; however, a sensitivity analyses were performed for the various filter and mode scenarios to confirm selected doses would remain optimized under those unique scenarios.

**TABLE 2:** Multiple linear regression of effluent flow rate, hemofilter type, CRRT mode on cefepime, and taniborbactam CL_TM_^*d*^

Drug, independent variable	Beta-coefficient	Std error	*P*-value
Cefepime			
Intercept	0.07	0.17	0.69
Effluent flow rate (L/h)^*a*^	0.86	0.05	<0.0001
Hemofilter^*b*^	0.25	0.08	0.04
CRRT mode^*c*^	0.66	0.09	<0.0001
Taniborbactam			
Intercept	0.10	0.14	0.62
Effluent flow rate (L/h)^*a*^	0.79	0.04	<0.0001
Hemofilter^*b*^	0.17	0.07	0.02
CRRT mode^*c*^	0.56	0.08	<0.0001

^*a*^ Effluent flow rates were assessed as continuous values in L/h.

^*b*^ ST150 = 0, HF1400 = 1.

^*c*^ CVVH = 0, CVVHD = 1.

^*d*^ CRRT, continuous renal replacement mode (i.e., CVVH vs CVVHD).

### Dose optimization

Monte Carlo simulation employing the population pharmacokinetic model from Phase 1 (healthy participants and patients with renal impairment) and 3 (urinary tract patients) with CL_CR_ of 60- ≥ 90 mL/min resulted in a mean cefepime area under the curve over 24 h (AUC_24h_) of 949 mg*h/L (+/− one SD range: 714–1184 mg*h/L) for a 2 g q8h dose, which matched the AUCs reported for the 287 subjects from the phase 1 and 3 trials (AUC day 1 geometric mean [%CV], 947 [23.8] mg*h/L for patients with CL_CR_ between 60 and 89 mL/min; and 812 [30.1] for patients with CL_CR_ ≥90 mL/min) (data on file, Venatorx Pharmaceuticals, Inc), thus demonstrating that the parameter estimates used for Monte Carlo simulation can recapitulate the observed AUCs from the clinical trial. The mean taniborbactam AUC_24h_ was 268 mg*h/L (+/− one SD range; 219–317) for a dose of 0.5 g q8h, which matched the day 1 AUC of the 287 patients (geometric mean [%CV], 318 [28] mg*h/L) for patients with CL_CR_ between 60 and 89 mL/min; and 263 [33.1] for patients with CL_CR_ ≥90 mL/min. These simulations provided the AUC limits used for the CRRT simulations.

Monte Carlo simulations were conducted at various effluent flow rates of 2–5 L/h in 1 L/h increments to predict cefepime and taniborbactam total AUC_24h_ and probability of target attainment (PTA) for free time above the MIC (*f*T >MIC) (for cefepime) and *f*AUC/MIC (for taniborbactam) following various dosing regimens (Tables 3 and 4). Additional Monte Carlo simulations were conducted as sensitivity analyses based on various CRRT scenarios using the full multivariate linear regression analyses (Tables S3 and S4).

**TABLE 3:** Monte Carlo simulation results: probability of attaining 1-log kill target of cefepime dosing regimens at different CRRT effluent flow rates

CRRT effluent flow rate (L/h)	Regimen (4 h infusion)	PTA^*a*^				C_min_ mg/L		Total AUC_24h_					*f*AUC_24h_
		4	8	16	32	Mean	SD	Mean	SD	CV	25th percentile	75th percentile	
Non-CRRT control^*b*^	2 g q8h	1	1	1	0.73	12.80	5.76	949	235	25%	764	1,103	785
2 L/h	2 g q8h	1	1	1	1	48	11	1,994	199	10%	1,851	2,123	1,649
	1 g q8h	1	1	1	0.9	24	5	997	99	10%	925	1,061	825
3 L/h	2 g q8h	1	1	1	1	32	8	1,558	128	8%	1,467	1,636	1,289
	1 g q8h	1	1	1	0.2	16	4	779	64	8%	733	818	644
	2 g q12h	1	1	1	0.9	12	5	1,041	85	8%	976	1,099	861
	1 g q12h	1	1	0.9	0.0	6	2	521	42	8%	488	550	430
4 L/h	2 g q8h	1	1	1	1	22	6	1,267	83	7%	1,211	1,321	1,048
	1 g q8h	1	1	1	0.0	11	3	633	41	7%	606	661	524
	2 g q12h	1	1	1	0.2	7	3	846	59	7%	804	885	700
	1 g q12h	1	1	0.2	0.0	4	2	423	29	7%	402	443	350
5 L/h	2 g q8h	1	1	1	1	16	5	1,075	60	6%	1,030	1,117	889
	1 g q8h	1	1	1	0.0	8	2	537	30	6%	515	558	444

^*a*^ PD target was 43.7% *f*T >MIC for cefepime.

^*b*^ Based on population pharmacokinetics provided by sponsor average of CrCl of 60, 90 and 120 mL/min.

**TABLE 4:** Monte Carlo simulation results: probability of attaining pharmacodynamic targets of taniborbactam dosing regimens at different CRRT effluent flow rates

CRRT effluent flow rate (L/h)	Regimen (4 h infusion)	PTA^*a*^				Total AUC_24h_					*f*AUC_24h_
		4	8	16	32	Mean	SD	CV	25th percentile	75th percentile	
Non-CRRT control^*b*^	0.5 g q8h	1	1	1	1	268	49	18%	231	297	221
2 L/h	0.5 g q8h	1	1	1	1	525	91	17%	452	593	461
	0.25 g q8h	1	1	1	1	263	46	17%	226	297	231
3 L/h	0.5 g q8h	1	1	1	1	410	54	13%	367	446	360
	0.25 g q8h	1	1	1	1	205	27	13%	184	223	180
	0.5 g q12h	1	1	1	1	278	38	14%	249	305	244
	0.25 g q12h	1	1	1	0.4	139	19	14%	125	153	122
4 L/h	0.5 g q8h	1	1	1	1	341	40	12%	309	368	299
	0.25 g q8h	1	1	1	0.9	170	20	12%	155	184	150
	0.5 g q12h	1	1	1	1	227	27	12%	206	246	199
	0.25 g q12h	1	1	1	0.0	113	13	13%	103	123	100
5 L/h	0.5 g q8h	1	1	1	1.00	289	29	10%	268	310	254
	0.25 g q8h	1	1	1	0.5	145	15	10%	134	155	127

^*a*^ PD target was *f*AUC/MIC ≥ 4 for taniborbactam.

^*b*^ Based on population pharmacokinetics provided by sponsor average of CrCl of 60, 90, and 120 mL/min.

## DISCUSSION

This study determined CL_TM_ of cefepime in combination with taniborbactam across different CRRT modalities with various effluent flow rates, point of fluid replacement, and using two different clinically used hemofilters in an *ex vivo* CRRT model. The study also evaluated the adsorption of each drug to the ST150 and HF1400 hemofilter circuits, two contemporary and commonly used filters in US hospitals. Both cefepime and taniborbactam exhibited high SA and SC and were essentially freely removed from the CRRT system. While the hemofilter type and CRRT mode were also significant predictors of CL_TM_, the effluent flow rate had the greatest influence on this estimate. Dosing regimen selection did not differ for specific hemofilter and CRRT mode scenarios; therefore, the optimal cefepime-taniborbactam dosing regimen could be determined based solely on the effluent flow rate. These findings are consistent with previous observations related to β-lactams, such as ceftolozane-tazobactam, imipenem-relebactam, cefiderocol, and sulbactam-durlobactam (14, 16–18).

The protein binding of each drug in the bovine plasma was assessed at the beginning and the end of the study. Cefepime resulted in 0% protein binding in bovine plasma; the absence of protein binding in our study is different from the protein binding of ~20% reported in humans (19). This negligeable protein binding value also varies from one of our recent *ex vivo* study for cefepime alone, which indicated an average protein binding of 20.8% ± 9.6% (20), and is likely related to different bovine blood batches used in these studies. Nonetheless, the absence of protein binding in the current study is concordant with SA and SC values of 1, as generally only free drug is available to pass through the hemofilters. For taniborbactam, protein binding in bovine plasma was 3.7% ± 3.8%, which is also lower than protein binding observed in some phase 1 trials (~12%) (Pharmacokinetic Report, PDS Project Number: VENATORX-008, data on file), but consistent with protein binding of 0%–0.7% observed during healthy volunteer bronchoscopy study (21). This is also consistent with the SA/SC for taniborbactam of ~1. Notably, cefepime dosing regimens derived from *ex vivo* studies and similar methodology to that conducted here were validated by observed exposures in patients receiving continuous veno-venous hemodiafiltration (CVVHDF) (20).

Based on the multiple linear regression analysis, the effluent flow rate, filter type, and CRRT mode were identified as significant factors affecting the CL_TM_ for both drugs (*P* < 0.05). While points of replacement dilution can also influence CL_TM_, this variable displayed high multicollinearity to the CRRT mode when assessed at 100% Pre-a and 50% pre-/50% post-, thus replacement was already factored into the CRRT mode during the statistical analyses. However, to simplify dosing regimens across different filter types and modes, we conducted additional multiple linear regression analyses focusing solely on the effluent flow rate. Sensitivity analysis was performed based on various CRRT scenarios, including both filter types and modes, and PTA and AUC_24h_ results continued to fall in acceptable ranges for the optimized doses selected with the simpler model based on effluent rate only (Tables S3 and S4). In previous studies on other β-lactams, only the effluent rate was found to statistically affect the CL_TM_ (14, 16, 17). This is also consistent with our *ex vivo* study that investigated cefepime alone and found that effluent flow rate was the predominant variable impacting cefepime CL_TM_ (20).

Two cefepime-taniborbactam dosing regimens were selected based on the results of these analyses. A dose of 1 g–0.25 g q8h administered as a 4 h infusion for effluent flow rates < 3.5 L/h provided a similar AUC_24h_ to a standard dosage in patients and healthy volunteers in phase 1 and 3 clinical trials and a high PTA at relevant MICs for both drugs. The full pneumonia dose of 2 g–0.5 g q8h as a 4 h infusion regimen provided similar AUC_24h_ and optimal PTA for effluent flow rates of 3.5 to 5 L/h. Additionally, 2 g–0.5 g q12h (4 h infusion) retained excellent PTA and similar AUC_24h_ at 3 and 4 L/h effluent rates and could be an option in that range (Tables 3 and 4). It should be noted that the pharmacokinetics of cefepime and taniborbactam used to perform these simulations were derived from healthy volunteers and patients who received 2 h instead of 4 h infusion. However, infusion duration will not impact pharmacokinetic parameters or AUC targets.

There are currently no clinical studies evaluating the combination of cefepime and taniborbactam in patients receiving CRRT. However, smaller studies have investigated cefepime pharmacokinetics in critically ill patients requiring CRRT support. In one study including patients with septic shock on CRRT support, the results indicated that the optimal dose of cefepime depends on the effluent flow rate. Specifically, a dose of 2 g q8h was necessary to provide adequate exposure at high effluent rates (22). In another prospective study, Philpott and colleagues examined 10 critically ill patients and recommended an extended infusion (2 g q8h as 4 h infusion) in critically ill patients receiving CRRT to retain a high probability of achieving pharmacodynamic targets for Enterobacterales and *Pseudomonas aeruginosa* (23).

The goal for the AUC values in the base model (i.e., non-CRRT control) was to select a dose for each compound that resulted in a mean exposure during the Monte Carlo simulation within 25th to 75th percentile of exposure observed in Phase 1 and Phase 3 patients who received the full dose of 2 g–0.5gq8h (as a 2 h infusion) (Tables 3 and 4). An exception was observed with taniborbactam when simulating an effluent rate of 3 L/h; the AUC based on the recommended dose (0.25 g q8h) was lower than the 25th to 75th percentile of the base model (205 vs 231–297 mg*h/L). This value, however, remains within the variability observed in Phase 1 and Phase 3 data and still achieved a high probability of obtaining pharmacodynamic thresholds. Additionally, q12h regimens at effluent rates of 3 L/h resulted in acceptable PTA and AUC values in the range of the 25th to 75th percentile, making these viable dosing options. In contrast, at an effluent rate of 4 L/h, the full dose (2 g q8h) resulted in a cefepime AUC at the upper end compared with the 25th and 75th percentiles of the base model used for comparison (1,267 vs.764–1,103 mg*h/L). It is important to note that the variability in our base model cohort was derived from Phase 1 and Phase 3 cUTI data. Published data for cefepime in critically ill patients has demonstrated greater variability in AUC, with the 25th and 75th percentiles ranging from 543 to 1,169 mg*h/L (24). Therefore, the full dose (2.5 g q8h) in our simulations would be recommended for effluent flow rates of ≥4 to 5 L/h. Our recommendations are based on assuming a linear CL_TM_ for both drugs as the effluent rate continues to rise. Nonetheless, additional studies are needed to confirm optimal dosing regimens for effluent rates well above 4 L/h.

While this study presents valuable insights, it is important to acknowledge its limitations. Even though we included different CRRT modes, two commonly used hemofilters, and typical effluent flow rates, the observations might not apply to other hemofilters or circuits made from other materials. Second, residual renal function was not accounted for due to the *ex vivo* nature of the model. Third, the simulations were conducted using only the 4 h infusion dose, as this is the infusion proposed for the treatment of HABP/VABP, and such patients are the most likely to require CRRT support. However, it’s worth noting that administering the drug over a 2 h infusion (as proposed for UTI) is unlikely to significantly affect dosage selection given the high PTA for cefepime and the absence of any infusion effect on the AUC. Finally, clinical pharmacokinetic studies are necessary to evaluate these dosing regimens to assess the impact of residual urine output and validate the proposed dosing exposures.

In summary, this study observed cefepime and taniborbactam to be efficiently cleared by both CVVH and CVVHD through ST150 and HF1400 hemofilters. The clearance of both drugs during CRRT was dependent primarily on the effluent flow rate. Several dosing regimens are proposed for pneumonia that can achieve optimized PTA and AUC values similar to those in non-CRRT patients with normal kidney function receiving the standard 2 g–0.5 g q8h (4 hour infusion) dosing regimen.

## MATERIALS AND METHODS

### *Ex vivo* CRRT

*Ex vivo* CRRT experiments were conducted as previously described by our group (18), utilizing a Prismaflex 7.2 control unit (Baxter Healthcare Corporation, Deerfield, IL, USA) in both CVVH and CVVHD modes. Two types of hemofilters were tested: a 1.4 m² polyarylethersulfone (PAES; Prismaflex HF1400 set; Baxter Healthcare Corporation) and a 1.5 m² acrylonitrile (AN69; Prismaflex ST150 set; Baxter Healthcare Corporation, REF # 213418). During CVVH at all rates, the replacement fluid was evaluated either as 100% pre-filter or as a 50% pre-filter and 50% post-filter. All experiments were conducted in duplicate for each mode, at flow rate, and with each filter type. Further details for the *ex vivo* CRRT procedures are described in the Supplemental Data.

### Study drug preparation and administration

Cefepime sodium 2 g/vial (Venatorx Therapeutics, lot 2L0885A42) was reconstituted with 10 mL sterile water for injection. Taniborbactam (VNRX-5133) 0.5 g/vial (Venatorx Therapeutics, lot B21090111) was reconstituted with 9.5 mL sterile water for injection to prepare stock solutions and frozen at −80°C. During each experiment, freshly thawed drug was injected in the blood reservoir to reach an approximate plasma target concentrations of 70 and 20 mg/L for cefepime and taniborbactam, respectively, which are the average maximum concentrations (*C*_max_) achieved in humans following a dose of 2 g–0.5 g q8h as a 4 h infusion. See Supplemental Data for additional details on study drug administration and sampling of the CRRT model.

### Degradation studies

To account for any degradation of cefepime or taniborbactam in the bovine whole blood over 2 hours, a control model was evaluated in triplicate as previously described (18). Degradation of less than 20% at any time point was considered negligible and not included in the adsorption calculation. The detailed explanation of the degradation experiments, and the equation used for calculation are listed in the Supplemental Data.

### Adsorption studies

The adsorption studies were conducted as previously described (18) to assess if cefepime or taniborbactam sequester to either hemofilter set (ST150 or HF1400). Adsorption of less than 20% was considered negligible and was not accounted for during Monte Carlo simulation. The details of the adsorption studies are described in the Supplemental Data.

### Bioanalytical procedures

Cefepime and taniborbactam concentrations were determined by Venatorx Pharmaceuticals Inc (Malvern, PA) using a validated LC-MS/MS method. A comprehensive description of the bioanalytical procedures is listed in the Supplemental Data. The BUN concentrations were determined using the kinetic methodology (UREAL; Roche Diagnostic Cobas 8000) at the Hartford Hospital Laboratory chemistry laboratory.

### Cefepime and taniborbactam transmembrane clearance

The sieving coefficient (SC, during CVVH) and saturation coefficient (SA, during CVVHD) of cefepime and taniborbactam were calculated as previously described (18) and used to calculate CL_TM_ for both drugs (Supplemental Data).

### Statistical analyses

Multiple linear regression (GraphPad Prism software version 10.0.3, La Jolla, CA, USA) was used to determine the influence of effluent flow rate, filter type, and CRRT mode on cefepime and taniborbactam CL_TM_.

### Monte Carlo simulations and dose recommendations

The goal of the simulations was to assess the probability of pharmacodynamic target attainment (PTA) and the achievable AUC exposures following dosing regimens to be included in the cefepime-taniborbactam drug label following different effluent dosages. Optimized CRRT dosing regimens were to achieve (i) similar or greater PTA for exposures that exceed what is needed to achieve 1-log of kill per the established pharmacodynamic targets for both cefepime (≥43.7% *f*T>MIC) and taniborbactam (*f*AUC/MIC ≥4) across the MIC range, and then, (ii) mean 24 hour total drug AUCs for the simulated regimen that fall within the 25th–75th percentiles for AUC exposures achieved with standard dosing in healthy volunteers and patients when corrected for creatinine clearance (CL_CR_). To simplify the process, the convenience of selecting proposed dosing regimens was considered, along with the goal of recommending as few dosing regimens as possible. A 1,000 patient Monte Carlo simulation (Pmetrics for R, Laboratory of Applied Pharmacokinetics and Bioinformatics, Los Angeles, CA) was performed using parameters obtained from a population pharmacokinetic analysis of 607 (*n* = 544 for cefepime) adult individuals from six phase 1 and one phase 3 clinical trials. The detailed procedure and assumptions for these Monte Carlo simulations are explained in the Supplemental Data.

## SUPPLEMENTAL MATERIAL

Supplemental material aac.00061-25-s0001.docx Supplemental Materials and Methods; Tables S1 to S4.

ASM does not own the copyrights to Supplemental Material that may be linked to, or accessed through, an article. The authors have granted ASM a non-exclusive, world-wide license to publish the Supplemental Material files. Please contact the corresponding author directly for reuse.
