# Supplementary material for: Ex vivo assessment and simulation to guide cefepime-taniborbactam dosing recommendations for patients receiving continuous renal replacement therapy
Source: Antimicrob Agents Chemother. 2025 May 5;69(6):e00061-25. doi: 10.1128/aac.00061-25 (PMC12135536; doi:10.1128/aac.00061-25)
Supplement: Supplemental material — s and Methods; Tables S1 to S4. [file aac.00061-25-s0001.docx]

Supplemental Data – March 31, 2025

***Ex Vivo* Assessment and Simulation to Guide Cefepime-Taniborbactam Dosing Recommendations for Patients Receiving** **Continuous Renal Replacement Therapy**

**MATERIALS AND METHODS**

***Ex Vivo CRRT***

At the start of each experiment, the Prismaflex circuit was primed with 0.9% sodium chloride, following the manufacturer's instructions. For each experiment, one liter of heparinized bovine blood (20 units/mL) from Lampire Biological Laboratories (Pipersville, PA, USA) was continuously stirred in a 2-liter beaker. The beaker was placed in a water bath set at 37°C, allowing the blood to circulate throughout the system for 10 minutes to enable equilibration before adding cefepime and taniborbactam. Throughout the experiments, the blood flow rate (Qb) was fixed at 200 mL/min, which is consistent with rates used clinically. CVVH replacement fluid (PrismaSOL® BGK 2/0; Baxter Healthcare Corporation) and CVVHD dialysate (PrismaSATE® BGK 2/0; Baxter Healthcare Corporation) flow rates of 2, 3, and 4 L/h were tested with each filter type.

***Study Drug Preparation and Administration***

The drug stocks were allowed to thaw prior to each *ex vivo* run. The drugs were then injected in the blood reservoir to reach an approximate plasma target concentration of 70 and 20 mg/L for cefepime and taniborbactam, which is the average maximum concentration (C_max_) achieved in humans following cefepime-taniborbactam 2g-0.5g q8h as a 4h infusion. Following drug injection, the system was allowed to equilibrate for 30 sec prior to sampling. Then, serial sampling from the central compartment (i.e., the beaker filled with 1L of blood) started (0 min), and at 10-, 30-, and 60-minutes following equilibration. Pre-filter blood samples, post-filter blood samples, and effluent samples were collected at 10, 30, and 60 minutes from the circuit ports. All blood samples were collected in sodium heparin vacutainer tubes (Becton Dickinson, Franklin Lakes, NJ, USA). Blood tubes were centrifuged at 2000x gravity for 10 min at 4°C, and the plasma was aliquoted into TrueNorth^®^ cryovials (Millipore Sigma, Rockville, MD, USA). The effluent samples were collected in TrueNorth^®^ cryovials (Millipore Sigma) directly. All plasma and effluent samples were frozen at -80°C until bioanalytical analysis.

***Protein Binding***

The purpose of these studies was to assess cefepime and taniborbactam protein binding in the bovine blood. Approximately 0.9 mL plasma samples obtained from the central blood compartment at 0 min and 60 min (for couple of runs) was aliquoted to Centrifree® Ultrafiltration Device (Merck Millipore Ltd, Tullagreen, Carrigtwohill, County Cork, Ireland) and centrifuged in a fixed-angle rotor at 1500 xg, 4°C, 45 to generate the protein free fraction (PFF). The resulting PFF and the aliquots of plasma samples were stored at -80°C until analysis for cefepime and taniborbactam concentrations in each matrix.

Protein binding was calculated as:

$$\% protein binding=100-\frac{concentration in PFF}{Concentration in plasma} * 100$$

***Degradation Studies***

The control model was a glass chemostat jar filled with 300 ml of bovine whole blood (i.e., the same matrix used in the *ex-vivo* CRRT system). The blood was inoculated with cefepime and taniborbactam as described above, and the chemostat jar was placed in a water bath at 37° C. Blood samples were collected at 0, 10, 20, 30, 45, 60, 90 and 120 minutes and processed similar to the *ex vivo* CRRT experiments.

Degradation was calculated as:

$$\%degradation=\frac{\left[ C abx 0 \right]-[C abx x]}{[C abx 0]}*100$$

where C_abx_ 0 is the concentration of cefepime or taniborbactam in the plasma at time zero, and C_abx_ x is the concentration of each drug in the control plasma at time x (e.g., 60 minutes).

***Adsorption Studies***

The *ex-vivo* CRRT model was modified to establish a closed-circuit system in which the effluent was redirected back to the central blood reservoir. To prevent the CRRT system from terminating due to weight imbalance, tap water was exogenously pumped into the effluent bag using a Masterflex^®^ Peristaltic Pump (Cole-Parmer, Vernon Hills, IL, USA) at the same rate as the effluent flow. During the same drug injection, bovine blood was supplemented with urea (Sigma-Aldrich, St. Louis, MO, USA) to achieve a final concentration of approximately 75 mg/dL. This was done to provide a control solute that accounts for drug dilution, as urea is known not to bind to the CRRT system. Blood urea nitrogen (BUN) was measured at the Hartford Hospital chemistry laboratory using a UREAL reagent assay on a COBAS 8000 analyzer (Roche Diagnostic, Indianapolis, IN) and multiplied by 2.14 to determine the endogenous urea content.

Blood samples were drawn from the central reservoir at 0, 10, 20, 30, 45, 60, 90 and 120 minutes and processed as described above, then frozen immediately at −80°C until analysis. A total of 8 adsorption experiments were performed incorporating various CRRT modes (CVVH and CVVHD) and filters (ST150 and HF1400) at a constant flow rate of 2 L/h. For CVVH mode, the replacement fluid was tested at 100% pre-filter only.

Adsorption was calculated as the difference between the total amount of the drug added to the system and the total amount recovered in the effluent and blood after 120 min. Dilution and degradation were incorporated into the adsorption calculation as follows at each time point:

$$\% diltuion factor=\frac{\left[ urea 0 \right]-[urea x]}{[urea 0]}*100$$

, where urea _0_ is urea concentration in the central blood compartment at time 0, and urea x is urea concentration in the blood at time x.

$$\% adsorption= \frac{\left[ C abx 0 \right]-[C abx x]}{[C abx 0]}*100-dilution factor \left( \% \right)-degradation (\%)$$

C_abx 0_ is the concentration of cefepime or taniborbactam in the pre-filter plasma at time zero, and C_abx x_ is the concentration of each agent in the pre-filter plasma at time x (e.g., 60 minutes).

***Bioanalytical Procedures***

Two bioanalytical methods for Taniborbactam (TAN) and Cefepime (FEP) were qualified by the discovery group of Venatorx Pharmaceuticals. One method used sodium heparin bovine plasma, and the other used saline. Both methods were performed on a Waters Acuity I-class liquid chromatography (LC) coupled with a Waters XEVO TQS Micro mass spectrometer (MS). Both methods utilized the same LC column (Waters Acquity HSS T3, 50×2.1mm, 1.8 µm) and mobile phase (0.1% formic acid in water as MP A and 0.1% formic acid in acetonitrile as MP B).  The transitions for TAN and FEP were 390.2 → 191 m/z and 481.28 → 167.18 m/z, respectively. Isotope-labeled internal standards were used, with transitions of 394.3 → 191 m/z for d4-TAN and 489.28 → 167.18 m/z for d8-FEP.

The gradient for the bovine plasma method used a constant flow rate of 0.6 mL/min. The gradient started by holding at 5% MP B for 0.3 minutes, then gradually increased to 95% MP B over 0.7 minutes. It was held at 95% MP B for 0.5 minutes, then rapidly decreased back to 5% MP B and held for an additional 0.5 minutes. The gradient for the saline method was slightly modified. With the same flow rate, the gradient started by holding at 3% MP B for 0.3 minutes, then increased to 20% MP B over 0.4 minutes. It was then raised to 95% MP B over the next 0.3 minutes, held for 0.5 minutes, and then rapidly decreased back to 3% MP B for an additional 0.5 minutes.

The bovine plasma method was qualified over a range of 50 to 50,000 ng/mL and 100 to 100,000 ng/mL for TAN and FEP, respectively. The intra-day accuracy for FEP and TAN were -13.1% to -2.2% bias and -12.5% to -0.9% bias, respectively; the inter-day accuracy for FEP and TAN were -12.5% to -7.5% bias and -12.4% to -2.4%, respectively. The intra-day precision for FEP and TAN were no more than 6.6% CV and 3.8% CV, respectively; the inter-day precision for FEP and TAN no more than 5.8% CV and 3.5% CV, respectively. Both analytes are stable in bovine plasma for up to 4 hours at 4 °C and up to 61 days at -80 °C. Furthermore, both analytes are stable for up to three freeze/thaw cycles and are stable for up to 3 hours in bovine whole blood.

The saline method was qualified over a range of 50 to 25,000 ng/mL and 100 to 50,000 ng/mL for TAN and FEP, respectively. The intra-day accuracy for FEP and TAN were -15.4% (only for lower limit of quantification) to 5.9% bias and -9.4% to 3.6% bias, respectively; the inter-day accuracy for FEP and TAN were -8.8% to 4.8% bias and -2.1% to 3.1%, respectively. The intra-day precision for FEP and TAN were no more than 10.2% CV and 4.7% CV, respectively: the inter-day precision for FEP and TAN no more than 9.7% CV and 7.3% CV, respectively. Both analytes are stable in saline for up to 5 hours at room temperature and up to 101 days at -80 °C. Furthermore, both analytes are stable for up to four freeze/thaw cycles.

***Cefepime and Taniborbactam Transmembrane Clearance***

The sieving coefficient (SC, during CVVH) and saturation coefficient (SA, during CVVHD) equations:

$$SC=\frac{2*Cuf}{Cpre+Cpost}$$

$$SA=\frac{2*Cdialysate}{Cpre+Cpost}$$

where C_uf_ is the concentration in the ultrafiltrate, C_pre_ is the concentration from the pre-filter sampling port, C_dialysate_ is the concentration in the dialysate, and C_post_ is the concentration from the post-filter sampling port.

CL_TM_ based on sieving coefficient (SC, during CVVH) and saturation coefficient (SA, during CVVHD) of cefepime-taniborbactam calculated as follows:

*CL_SC_ (CVVH)=* $\frac{SC * Quf * Qb}{Qb + Qrep}$

*CL_SA_ (CVVHD)=* $SA *Q$*d*

, where Q_uf_ is the ultrafiltration flow rate, Q_b_ is the blood flow rate, Q_rep_ is the pre-replacement fluid rate, and Q_d_ is the dialysate flow rate.

***Monte Carlo Simulations and Dose Recommendations***

To obtain these standard deviation ranges, a 1000 patient Monte Carlo simulation (Pmetrics for R, Laboratory of Applied Pharmacokinetics and Bioinformatics, Los Angeles, CA) was performed using parameters obtained from a population pharmacokinetic analysis of 607 (n=544 for cefepime) adult individuals from six phase 1 and one phase 3 clinical trials (ICPD Report Number: ICPD 00443-1; provided by Venatorx Pharmaceuticals, Inc.). The simulations included the standard dosing regimen for pneumonia (2g-0.5g q8h as 4h infusion) for patients with a CL_CR_ divided into three distinct renal function categories: 60, 90, and 120 mL/min. To introduce variability in the CL_CR_, a coefficient of variation (CV) of 15% was applied. For the other pharmacokinetic parameters, variability was derived from the ICPD report (ICPD 00443-1), which indicated CVs of 53.8% for Vc and 6.26% for Vp for cefepime. Additionally, a variability of 30% was assumed for Q for both drugs, while the variability around Vp for taniborbactam was also assumed to be 30%. The total drug AUCs_24h_ were calculated by trapezoidal rule for each simulated patient and compared with the AUC_day 1_ reported by ICPD for use as a target reference for CRRT total drug AUC_24h_ exposures. The PTA and AUC results for the three simulated populations were averaged to acquire the target exposures for the control (non-CRRT) population.

The average cefepime parameters used in the Monte Carlo simulation for healthy volunteers and patients with CL_CR_ of 60, 90, and 120 mL/min were as follows:

CL (L/h) _=_ 4.75*(BCLCR/77)^0.696^*(1-0.613) ^ESRD^*(1+0.281) ^cUTI/AP^

a) CL_CR_ of 60 ml/min: 4.75 x (CLCR/77)^0.696^ x1.281) = 5.1 L/h

b) CL_CR_ of 90 ml/min: 4.75 x (CLCR/77)^0.696^ x1.281) = 6.78 L/h

c) CL_CR_ of 120 ml/min: 4.75 x (CLCR/77)^0.696^ x1.281) = 8.29 L/h

Vc (L), 8.49

Q (L/h), 5.94

Vp (L), 6.26

The average taniborbactam parameters used in the Monte Carlo simulation for healthy volunteers and patients with CL_CR_ of 60, 90, and 120 mL/min were as follows:

CL (L/h) _=_ 4.81*(BCLCR/82)^0.733^*(1-0.689) ^ESRD^

a) CL_CR_ of 60 ml/min: 4.81 x (CLCR/82)^0.733^ = 3.83 L/h

b) CL_CR_ of 90 ml/min: 4.81 x (CLCR/82)^0.733^ = 5.15 L/h

c) CL_CR_ of 120 ml/min: 4.81 x (CLCR/82)^0.733^ = 6.35 L/h

Vc (L), 12.2

Q (L/h), 3.49

Vp (L), 6.2

For simulations at different effluent flow rates, renal clearance (CL_R_) from the population PK model was substituted with the CL_TM_ obtained from the *ex vivo* studies. Cefepime and taniborbactam non-renal clearance (CL_NR_) (derived from patients with varying degrees of renal impairment, PDS Project Number: VENATORX-008) were 0.87 and 0.77 L/h, respectively. A 30% CV was added to the CL_NR_ values to account for uncertainty since the CL_NR_ in patients with AKI and receiving CRRT is undefined. Total cefepime and taniborbactam clearance was the sum of the CL_NR_ and the CL_TM_. All other parameter estimates remained similar to the baseline population as described above. The dose of cefepime-taniborbactam 2g-0.5g q8h as a 4h infusion was simulated across fixed effluent rates of 2, 3, 4, and 5 L/h. Additional dosing regimens of 1g-0.25g and 2g-0.5g q12h as 4h infusions was simulated across select effluent rates (2, 3, and 4 L/h) based on the exposure results from the standard dose at that rate. During all simulations, the protein binding of cefepime and taniborbactam were fixed at 17.3% and 12.2%, respectively. These values were derived from PDS Project Number: VENATORX-008.

In addition, multiple simulations were conducted using CL_TM_ calculations across various CRRT scenarios, including different filters and modes. These simulations were performed whenever an independent variable was found to statistically impact the CL_TM_. The goal was to determine whether different dosing recommendations might be necessary based on the filter type, CRRT mode, or replacement fluid.

**RESULTS**

**Table S1.** Summary of cefepime and taniborbactam percent degradation over time. Data presented as mean ± SD for three individual runs. Positive integers indicate reductions from zero.

|  | Degradation from Baseline (%) | |
| --- | --- | --- |
| Time (min) | Cefepime | Taniborbactam |
| 10 | 4.83 ± 8.13 | 5.47 ± 7.01 |
| 20 | 3.42 ± 4.93 | 4.51 ± 3.03 |
| 30 | 2.74 ± 4.38 | 4.80 ± 3.84 |
| 45 | 4.30 ± 5.13 | 5.81 ± 3.78 |
| 60 | 5.94 ± 5.20 | 6.39 ± 0.32 |
| 90 | 4.69 ± 4.30 | 4.83 ± 2.94 |
| 120 | 6.91 ± 6.16 | 7.14 ± 2.93 |

**Table S2**. Cefepime and taniborbactam percent adsorption across different CRRT modes and hemofilters.

|  |  | Adsorption (%) | |
| --- | --- | --- | --- |
| CRRT mode | Filter | Cefepime | Taniborbactam |
| CVVH | ST150 | 10.13 ± 2.84 | 11.15 ± 3.89 |
| CVVH | HF1400 | 14.33 ± 4.59 | 13.85 ± 4.38 |
| CVVHD | ST150 | 8.90 ± 3.88 | 10.07 ± 4.38 |
| CVVHD | HF1400 | 13.98 ± 4.40 | 11.86 ± 3.69 |

##

**Table S3**. Sensitivity Analyses: Probability of attaining 1-log kill target of cefepime dosing regimens at different CRRT effluent flow rates, CRRT modes, and filter types. PD target was 43.7% *f*T>MIC for cefepime.

| CRRT effluent flow rate (L/h), mode, filter | Regimen (4h infusion) | PTA | | | | | | | C_min_ mg/L | | | Total AUC_24h_ | | | | | | | | *f*AUC_24h_ |  |
| --- | --- | --- | --- | --- | --- | --- | --- | --- | --- | --- | --- | --- | --- | --- | --- | --- | --- | --- | --- | --- | --- |
|  |  | **4** | | **8** | | **16** | | **32** | **mean** | **SD** | | **mean** | | **SD** | | **CV** | **25^th^** | **75^th^** | |  |  |
| 3, CVVH, ST150 | 2g q8h | 1 | | 1 | | 1 | | 1 | 37 | 9 | | 1703 | | 155 | | 9% | 1591 | 1801 | | 1408 |  |
|  | 1g q8h | 1 | | 1 | | 1 | | 0.46 | 19 | 4 | | 851 | | 78 | | 9% | 796 | 901 | | 704 |  |
| 3, CVVH, HF1400 | 2g q8h | 1 | | 1 | | 1 | | 1 | 33 | 8 | | 1601 | | 136 | | 8% | 1503 | 1685 | | 1324 |  |
|  | 1g q8h | 1 | | 1 | | 1 | | 0.23 | 17 | 4 | | 800 | | 68 | | 8% | 752 | 843 | | 662 |  |
| 3, CVVHD, ST150 | 2g q8h | 1 | | 1 | | 1 | | 1 | 28 | 7 | | 1447 | | 111 | | 8% | 1368 | 1516 | | 1196 |  |
|  | 1g q8h | 1 | | 1 | | 1 | | 0.03 | 14 | 4 | | 723 | | 55 | | 8% | 684 | 758 | | 598 |  |
| 3, CVVHD, HF1400 | 2g q8h | 1 | | 1 | | 1 | | 1 | 25 | 6 | | 1357 | | 99 | | 7% | 1286 | 1421 | | 1122 |  |
|  | 1g q8h | 1 | | 1 | | 1 | | 0.0 | 12 | 3 | | 679 | | 49 | | 7% | 643 | 710 | | 561 |  |
| 4, CVVH, ST150 | 2g q8h | 1 | | 1 | | 1 | | 1 | 25 | 7 | | 1368 | | 99 | | 7% | 1294 | 1432 | | 1131 |  |
|  | 1g q8h | 1 | | 1 | | 1 | | 0.0 | 13 | 3 | | 684 | | 50 | | 7% | 647 | 716 | | 566 |  |
| 4, CVVH, HF1400 | 2g q8h | 1 | | 1 | | 1 | | 1 | 23 | 6 | | 1301 | | 95 | | 7% | 1227 | 1368 | | 1076 |  |
|  | 1g q8h | 1 | | 1 | | 1 | | 0.0 | 12 | 3 | | 651 | | 48 | | 7% | 613 | 684 | | 538 |  |
| 4, CVVHD, ST150 | 2g q8h | 1 | | 1 | | 1 | | 1 | 20 | 6 | | 1197 | | 80 | | 7% | 1134 | 1254 | | 990 |  |
|  | 1g q8h | 1 | | 1 | | 1 | | 0.0 | 10 | 3 | | 599 | | 40 | | 7% | 567 | 627 | | 495 |  |
| 4, CVVHD, HF1400 | 2g q8h | 1 | | 1 | | 1 | | 1 | 18 | 5 | | 1135 | | 68 | | 6% | 1084 | 1180 | | 939 |  |
|  | 1g q8h | 1 | | 1 | | 1 | | 0.0 | 9 | 3 | | 567 | | 34 | | 6% | 542 | 590 | | 469 |  |
| 5, CVVH, ST150 | 2g q8h | 1 | | 1 | | 1 | | 1 | 18 | 5 | | 1142 | | 68 | | 6% | 1092 | 1188 | | 945 |  |
|  | 1g q8h | 1 | | 1 | | 1 | | 0.0 | 9 | 3 | | 571 | | 34 | | 6% | 546 | 594 | | 472 |  |
| 5, CVVH, HF1400 | 2g q8h | 1 | | 1 | | 1 | | 1 | 17 | 5 | | 1092 | | 65 | | 6% | 1045 | 1138 | | 903 |  |
|  | 1g q8h | 1 | | 1 | | 1 | | 0.0 | 8 | 3 | | 546 | | 32 | | 6% | 523 | 569 | | 451 |  |
| 5, CVVHD, ST150 | 2g q8h | 1 | | 1 | | 1 | | 1 | 14 | 5 | | 1018 | | 56 | | 6% | 978 | 1057 | | 841 |  |
|  | 1g q8h | 1 | | 1 | | 1 | | 0.0 | 7 | 2 | | 509 | | 28 | | 6% | 489 | 529 | | 421 |  |
| 5, CVVHD, HF1400 | 2g q8h | 1 | | 1 | | 1 | | 1 | 13 | 4 | | 975 | | 50 | | 5% | 938 | 1009 | | 806 |  |
|  | 1g q8h | 1 | 1 | | 1 | | 0.0 | | 6 | | 2 | | 487 | | 25 | 5% | 469 | | 504 | 403 | |

**Table S4**. Sensitivity Analyses: Probability of attaining 1-log kill target of taniborbactam dosing regimens at different CRRT effluent flow rates, CRRT modes, and filter types. PD target was *f*AUC/MIC ≥4 for taniborbactam.

| CRRT effluent flow rate (L/h), mode, filter | Regimen (4h infusion) | PTA | | | | Total AUC_24h_ | | | | | *f*AUC 24h |
| --- | --- | --- | --- | --- | --- | --- | --- | --- | --- | --- | --- |
|  |  | **4** | **8** | **16** | **32** | **mean** | **SD** | **CV** | **25^th^** | **75^th^** |  |
| 3, CVVH, ST150 | 0.5g q8h | 1 | 1 | 1 | 1 | 445 | 65 | 15% | 395 | 491 | 391 |
|  | 0.25g q8h | 1 | 1 | 1 | 1 | 223 | 32 | 15% | 197 | 245 | 195 |
| 3, CVVH, HF1400 | 0.5g q8h | 1 | 1 | 1 | 1 | 432 | 61 | 14% | 385 | 476 | 380 |
|  | 0.25g q8h | 1 | 1 | 1 | 1 | 216 | 31 | 14% | 192 | 238 | 190 |
| 3, CVVHD, ST150 | 0.5g q8h | 1 | 1 | 1 | 1 | 388 | 50 | 13% | 349 | 424 | 341 |
|  | 0.25g q8h | 1 | 1 | 1 | 0.99 | 194 | 25 | 13% | 175 | 212 | 170 |
| 3, CVVHD, HF1400 | 0.5g q8h | 1 | 1 | 1 | 1 | 369 | 45 | 12% | 334 | 402 | 324 |
|  | 0.25g q8h | 1 | 1 | 0.9 | 0.98 | 184 | 22 | 12% | 167 | 201 | 162 |
| 4, CVVH, ST150 | 0.5g q8h | 1 | 1 | 1 | 1 | 360 | 45 | 12% | 326 | 392 | 316 |
|  | 0.25g q8h | 1 | 1 | 1 | 0.95 | 180 | 23 | 12% | 163 | 196 | 158 |
| 4, CVVH, HF1400 | 0.5g q8h | 1 | 1 | 1 | 1 | 352 | 43 | 12% | 319 | 382 | 309 |
|  | 0.25g q8h | 1 | 1 | 1 | 0.93 | 176 | 21 | 12% | 160 | 191 | 155 |
| 4, CVVHD, ST150 | 0.5g q8h | 1 | 1 | 1 | 1 | 322 | 36 | 11% | 295 | 347 | 283 |
|  | 0.25g q8h | 1 | 1 | 1 | 0.77 | 161 | 18 | 12% | 147 | 174 | 141 |
| 4, CVVHD, HF1400 | 0.5g q8h | 1 | 1 | 1 | 1 | 309 | 33 | 11% | 284 | 332 | 271 |
|  | 0.25g q8h | 1 | 1 | 0.97 | 0.67 | 154 | 17 | 12% | 142 | 166 | 135 |
| 5, CVVH, ST150 | 0.5g q8h | 1 | 1 | 1 | 1 | 304 | 31 | 10% | 279 | 327 | 267 |
|  | 0.25g q8h | 1 | 1 | 1 | 0.62 | 152 | 16 | 10% | 139 | 164 | 133 |
| 5, CVVH, HF1400 | 0.5g q8h | 1 | 1 | 1 | 1. | 296 | 30 | 10% | 274 | 318 | 260 |
|  | 0.25g q8h | 1 | 1 | 1 | 0.55 | 148 | 15 | 10% | 137 | 159 | 130 |
| 5, CVVHD, ST150 | 0.5g q8h | 1 | 1 | 1 | 1 | 274 | 26 | 9% | 255 | 293 | 241 |
|  | 0.25g q8h | 1 | 1 | 1 | 0.26 | 137 | 13 | 9% | 128 | 146 | 120 |
| 5, CVVHD, HF1400 | 0.5g q8h | 1 | 1 | 1 | 1 | 265 | 24 | 9% | 247 | 282 | 232 |
|  | 0.25g q8h | 1 | 1 | 0.97 | 0.15 | 132 | 12 | 9% | 123 | 141 | 116 |
